# Reviews and Book Notices

**Published:** 1877-06

**Authors:** 


### Reviews and Book Notices.

A Manual of General Pathology for the use of Students and Practitioners of Medicine. By *Ernst Wagner*, *M. D.*, Professor of General Pathology and Pathological Anatomy in the University of Leipzig, etc. Translated from the sixth German edition by *John Van Duyn*, *A. M.*, *M. D.*, etc., and *E. C. Seguin*, *M. D.*, etc. New York: William Wood & Co. 1876.

In no part of the world are the fruits of the German Laboratories more eagerly gathered up, more thoroughly sifted and more generally utilized, than right here in America. Nor is this to be wondered at. We are essentially a utilitarian people, valuing things and ideas according to the measure of their adaptability to our practical wants and necessities. From precisely this utilitarian standpoint do we estimate the value of such books as those of Virchow, Billroth, Rindfleisch, Gluge, and the one before us. The German Medical Schools are in some sort great analytical laboratories; the German mind and mode of thought is essentially analytical; the German patiently and laboriously picks into pieces whatever comes before him, classifies and carefully labels the component parts, but rarely thinks of re-combining the integers he has dissevered. On the other hand, the American Medical School—so far as it has assumed a distinctive character at all —is synthetical; to build up, improve, utilize and economize is the American idea. How do German books look from this American standpoint?

Wagner's "Manual of Pathology," first issued in Germany in 1862, is now (January, 1876,) for the first time presented in its English dress. It is an event which merits more than a passing notice, especially in view of the intense activity which now prevails, especially on the continent of Europe in every department of pathological research. The general plan of the work is as follows: Part First—General Nosology; Part Second—General Ætiology; Part Third—General Pathological Anatomy and Physiology, and Part Fourth—Pathology of the Blood.

As we are somewhat limited as regards space, we shall pass rapidly over the parts relating to "General Nosology" and "General Ætiology," and devote our attention mainly to the parts treating of "General Pathological Anatomy and Physiology" and the "Pathology of the Blood."

We are pained at the threshhold of this book, to find that the translators have made Prof. Wagner responsible for such a barbarous word as "diagnosed" (page 2); it is hardly fair to make the helpless German professor the father of such an uncouth child.

On the same page we find this general statement; "the disturbances which constitute disease" are: Anatomnico-Pathological, (that is to say histological), or, Chemico-Pathological, or Functional. But with respect to the so-called "functional" diseases, the author makes this most wise observation: "Functional or symptomatic (or dynamical) disorders are those in which we have, as yet, been unable to detect any material lesion. This, for example, is often the case in pain and convulsions. And yet in these conditions we must admit the existence of *delicate material alterations, which are not*

*recognizable with our present means of investigations.*" (Italics are our own). The idea that disorders of function present no material changes, and are therefore destitute of interest to the histologist, ought to be abandoned at once and forever.

Turning to "Part Second," we find a very comprehensive, and, in the main, admirable essay, occupying more than one hundred pages, upon "*General Ætiology.*" It seems to us, however, that the general tone of positiveness which characterizes this part of the work, is scarcely warranted by the existing state of that branch of medical science which relates to the causes of disease. And we are the more surprised at this because the very first sentence of this chapter contains these words; "Ætiology, or the knowledge of the causes of disease, is one of the weakest chapters in pathology"—a statement which every student of pathology is unfortunately compelled to endorse. If there is a single department of medical science concerning which our knowledge is so notably defective as to make it especially prominent on that account, it is the department of Ætiology; hence writers and teachers should exercise a studious caution in the expression of their opinions. But on the other hand, it is very encouraging to witness the great activity now manifested in all parts of the world, in the investigation of the causes of disease. Not very many years hence, it will be "in order" for an author to express his opinions upon this point with some degree of assurance that it amounts to something more than mere guess-work.

Part Third—"General Pathological Anatomy and Physiology" occupies three hundred and sixty nine pages, making something more than half the entire work. We are glad to observe that Prof. Wagner so pointedly recognizes the fact that Physiology and Pathology are most intimately related—that in truth there is no dividing line between them. Those of us who are engaged in teaching Pathology, know only too well how difficult it is to convey to the mind of the student, the idea that there is no abrupt and strongly marked boundary line between health and disease; that the same tissues and the same vital elements are involved, whether the various functions of the economy be carried on healthily (Physiologically) or unhealthily (Pathologically). Indeed, quite too often the first and most difficult duty of the teacher, is to disabuse the mind of the beginner in medical science of the notion that "disease" is a sort of lawless invader—a something entirely *sui generis*, which takes possession of the system and proeeeds to do various acts of violence, unless evicted by some sort of a therapeutical mitralleuse which the physician selects from among his manifold resources, and discharges at the offender. But it is a hopeful sign of progress when text-books—which are generally behind rather than in advance of aggressive professional opinion—take cognizance of the fact that Physiology and Pathology have a common point of departure.

The part of Prof. Wagner's book now under consideration seems to us to possess great value. It is built upon an excellent plan, and the plan is successfully carried out. It is not loaded down with the debris of long-forgotten theories, or water-logged by being forced to carry all the crude and half digested notions which in this age of ceaseless activity must necessarily encumber the precincts of medical science. Good judgment has been exercised both in what is said, and what is left unsaid. We commend this part of the work to the student and young practitioner, because it is in the main worth studying and worth remembering. It is refreshing to find the latest discoveries concerning the circulation in the lymphatic system; the spontaneous movements of the white blood corpuscles, or "leucocytes;" and the origin, function, and destiny of fibrin so clearly and admirably stated. These three questions, (more especially the two last) have been a stumbling block to most teachers of pathology for years past. But Prof. Wagner simply accepts the doctrines which now seem to be established, states them with impartial frankness, and then leaves them to take care of themselves. He does not seem to be hampered by preconceived hobbies, which he feels bound to bolster up by "arguing the case." The present somewhat diverse doctrines concerning inflammation and the origin of pus corpuscles, are clearly and impartially stated. Part fourth—the "Pathology of the Blood"—is the concluding portion of the work. Its merits are many; its faults are comparatively few. It is impossible for one author to cover so wide a range of subjects as Prof. Wagner attempts to include in his book; moreover, it is equally impossible to crowd into a single volume—unless it be of gigantic proportions—all that ought to be said even in a "Manual" of "General Pathology;" but our author has probably come as near accomplishing the impossible as any one could.

We do not like the chopped style, or the angular, juiceless sentences with which the book abounds; we detest the small type and closely crowded lines; but the plan of the work renders both a matter of necessity.

The translators have done their work admirably well. It is a rare luxury to find a German work translated into readable English. Many of the German books which have appeared in English dress make a dreadfully stiff and awkward appearance.

Perhaps "Rindfleich's Pathological Histology" wears the most slovenly and ill-fitting English suit of any book yet "translated." It is doubtful if Rindfleisch would recognize his own ideas in the solemn and ghostly garb which they are compelled to wear since we tried to naturalize them. The cheerful and sprightly pages of Flavius Josephus are fascinating—we had almost said side-splitting—in comparison with the monkish austerity of the English edition of that most admirable book, "Rindfleisch's Pathological Histology."

The translation of "Frey's Compendium of Histology" is almost as bad; it is needlessly unreadable and inexcuseably unenjoyable.

We turn with pleasure to the English—or American—edition of "Wagner's Manual;" the translators seem to have done their work with severe accuracy, but they did not forget that their task involved the translation of *ideas* as well as words. We believe Prof. Wagner's work will be widely read in this country; certainly nothing better of its kind is now attainable.

